# Contextual and Perceptual Brain Processes Underlying Moral Cognition: A Quantitative Meta-Analysis of Moral Reasoning and Moral Emotions

**DOI:** 10.1371/journal.pone.0087427

**Published:** 2014-02-04

**Authors:** Gunes Sevinc, R. Nathan Spreng

**Affiliations:** 1 Laboratory of Brain and Cognition, Department of Human Development, Cornell University, Ithaca, New York, United States of America; 2 Human Neuroscience Institute, Cornell University, Ithaca, New York, United States of America; 3 Department of Neurosciences, Institute for Medical Research, Istanbul University, Istanbul, Turkey; 4 Department of Humanities and Social Sciences, Faculty of Arts and Sciences, Yildiz Technical University, Istanbul, Turkey; Bellvitge Biomedical Research Institute-IDIBELL, Spain

## Abstract

**Background and Objectives:**

Human morality has been investigated using a variety of tasks ranging from judgments of hypothetical dilemmas to viewing morally salient stimuli. These experiments have provided insight into neural correlates of moral judgments and emotions, yet these approaches reveal important differences in moral cognition. Moral reasoning tasks require active deliberation while moral emotion tasks involve the perception of stimuli with moral implications. We examined convergent and divergent brain activity associated with these experimental paradigms taking a quantitative meta-analytic approach.

**Data Source:**

A systematic search of the literature yielded 40 studies. Studies involving explicit decisions in a moral situation were categorized as active (n = 22); studies evoking moral emotions were categorized as passive (n = 18). We conducted a coordinate-based meta-analysis using the Activation Likelihood Estimation to determine reliable patterns of brain activity.

**Results & Conclusions:**

Results revealed a convergent pattern of reliable brain activity for both task categories in regions of the default network, consistent with the social and contextual information processes supported by this brain network. Active tasks revealed more reliable activity in the temporoparietal junction, angular gyrus and temporal pole. Active tasks demand deliberative reasoning and may disproportionately involve the retrieval of social knowledge from memory, mental state attribution, and construction of the context through associative processes. In contrast, passive tasks reliably engaged regions associated with visual and emotional information processing, including lingual gyrus and the amygdala. A laterality effect was observed in dorsomedial prefrontal cortex, with active tasks engaging the left, and passive tasks engaging the right. While overlapping activity patterns suggest a shared neural network for both tasks, differential activity suggests that processing of moral input is affected by task demands. The results provide novel insight into distinct features of moral cognition, including the generation of moral context through associative processes and the perceptual detection of moral salience.

## Introduction

Neuroimaging research has provided substantial insight into the biological processes involved in moral cognition. Investigations into the neural correlates of morality have centered upon moral reasoning on the one hand, and morally-determined affective response, or moral emotions, on the other [1]–[5]. Studies examining these two aspects of moral cognition have utilized a variety of tasks that include an active judgment of scenarios depicting sophisticated moral dilemmas, or passive viewing of pictures or sentences describing immoral actions. Moral processes that are investigated using these tasks involve deliberative reasoning and/or rapid, affectively guided responses [6]–[8]. While both emotion and social cognition are integral to moral reasoning [9], [10], the processing of moral input and its neural correlates may differ depending on specific task demands [8]. In the present study, we determined convergent and divergent patterns of activity underlying these different task categories.

Experimental paradigms typically used to study moral cognition emphasize deliberative explicit cognitive processes, or affective perceptual processes. Explicit moral judgment tasks isolate neural activity associated with reflective forms of moral judgments that leads to an active decision in a morally ambiguous situation [11], [12]. In the typical trolley dilemma, participants are asked to make an explicit moral decision about whether they would hit a switch to save the life of five people at the expense of killing one [1]. The activity during these tasks is contrasted with activity during non-moral judgments, such as about whether to travel by bus or by train given certain time constraints. In contrast, moral emotion paradigms isolate neural activity associated with an automatic response elicited by the perception of a morally salient stimulus [13]–[15]. For example, participants are asked to make an indoor/outdoor judgment looking at a photograph depicting a moral transgression versus looking at an unpleasant social scene without moral content. In these tasks, the morally salient features of the scenario are incidental to the task demands [16] and stimuli are typically contrasted with neutral or other emotional (e.g. unpleasant non-moral) stimuli. Investigation of overlapping and divergent patterns of activity associated with these tasks, that presumably reflect different forms of moral processing, may provide new insight for a unified neural framework of moral cognition.

Overlap between brain activity during judgments of moral dilemmas and regions of the default network have been observed [12], [17]. The default network plays an active role in cognition and supports internally-generated thought [18]. These modes of thought include episodic and semantic memory, inferring the mental state of others, self-referential processing, simulation of possible events, scene construction and contextual associations [18]–[23]. These processes relate to moral cognition, as the moral quality of an event involves spontaneous or deliberate interpretation beyond the immediate perceptual experience. For instance, both active moral judgments and passive viewing of immoral scenes involve social contexts, where mentalizing about the protagonists will take place [24]. Additionally, simulation may be necessary for more deliberative forms of moral reasoning, such as a moral dilemma. In contrast however, more rapid or automatic forms of moral processing might be relevant for situations where elaboration is unnecessary or inefficient.

Here, we investigated reliable patterns of brain activity during performance on a diversity of moral cognition tasks using quantitative meta-analysis. From a comprehensive search of the literature, we categorized neuroimaging studies of morality based on the presence or absence of an explicit moral judgment. Reliable activity patterns were determined for explicit moral judgment tasks (i.e. “active”) and for tasks using passive exposure to morally laden stimuli (i.e. “passive”). We performed contrast and conjunction analyses to assess convergent and divergent areas of activation. We hypothesized that both active and passive tasks would result in increased activity in the default network, reflecting deliberative or spontaneous mentalizing about the actors involved and contextual processing of the information. For active tasks, we predicted more robust engagement of default network structures in support of elaborate constructive processes, such as scene construction. For passive tasks, we predicted unique activity in neural areas associated with emotional and visual information processing such as the amygdala and ventral temporal regions, reflecting the heightened perceptual and emotional processing of morally salient stimuli.

## Methods

**Figure 1:**
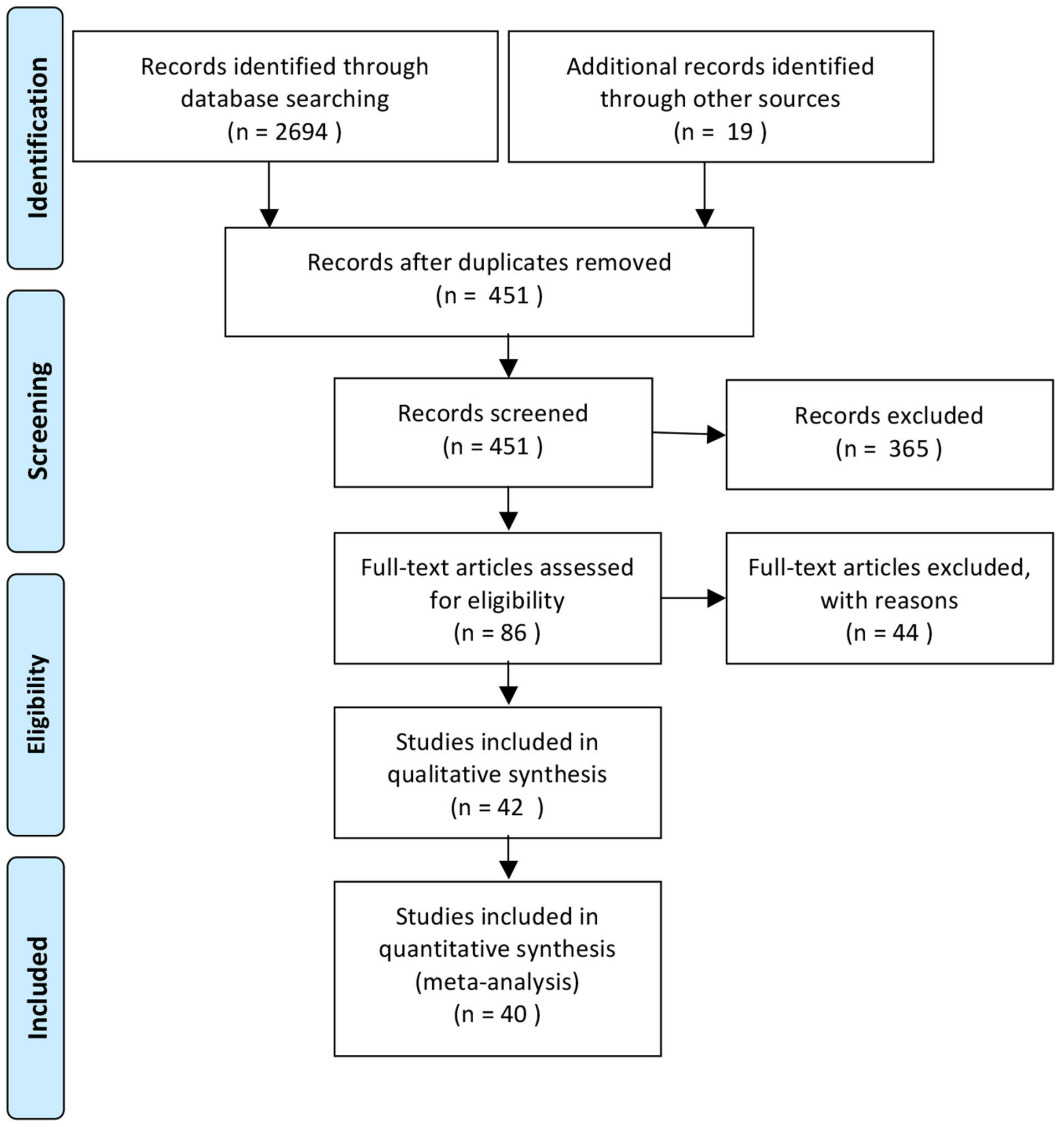
Flow diagram for literature search (Flow Diagram S1).

To identify neuroimaging studies related to morality, we conducted a systematic literature search of Pubmed, PsycINFO and Web of Science using the following keywords: “moral*”; AND “fMRI” <OR> “functional magnetic resonance imaging” <OR> “brain imaging” <OR> “positron emission tomography” <OR> “PET; AND “human”. We refined the results of this search using additional filters provided by each database. Than those records were screened a) based on the title and journal information, b) based on the information available on the abstract, c) based on full-text screening respectively. Selection criteria involved: (1) inclusion of a healthy adult participant group, (2) investigation of morality related activation using moral vs. non-moral contrast and (3) reporting of voxelwise, whole-brain data. See figure 1 for details regarding the literature search flow.

Studies only reporting results within different domains of morality were excluded, such as deontological judgment vs. utilitarian, or counterintuitive vs. intuitive moral judgments [25], because they don’t fulfill the second inclusion criterion of having a moral vs. non-moral contrast. Studies that reported activation foci in ways other than 3D coordinate space were excluded (e.g. ROI analysis) as they are not compatible with the activation likelihood estimation (ALE) analysis approach. For articles that reported both between- and within-group contrasts, we included foci from appropriate within-group contrasts. Moreover, as a result of absence of criteria for what makes a stimuli-set moral, we included only those studies, in which authors made an explicit claim that the stimuli had a moral content. From the studies that reported multiple contrasts using the same participants, we selected the contrast that is most relevant to the moral domain. Due to lack of a widely accepted definition of morality, we relied on authors’ claim to determine relevance. When multiple contrasts were provided, instead of more specific contrasts, the contrast revealing the broadest conception of moral cognition such as ‘moral (care, justice)>neutral (strategic, tactical)’ was selected. We searched the references of all included studies and recent review papers for additional studies. In total, this search yielded 40 articles for the ALE analyses (39 studies using fMRI and 1 study using PET) including data from 772 participants and 399 foci. A complete list of contrasts can be found in the Table 1 and a complete list of studies included can be found in the appendix (Appendix S1).

**Table 1:** Studies included in meta-analysis Active Category and Studies included in meta-analysis Passive Category.

First Author	Year		n		Foci		Contrast	Active
Avram	2013		16		8		moral judgment>aestethic judgment	
Bahnemann	2010		25		11		moral judgment >physical norm judgment	
Borg	2006		24		7		moral>nonmoral judgment	
Borg	2011		26		37		moral>nonmoral judgment	
Chiong	2013		16		3		personal moral>nonmoral jugdment	
FeldmannHall	2012		14		9		moral>nonmoral decision	
FeldmannHall	2013		35		6		moral>nonmoral (difficult) decision	
Harada	2009		18		9		moral>nonmoral judgment	
Harenski	2008		28		10		moral>nonmoral judgment	
Harrison	2008		22		5		moral>rest	
Hayashi	2010		12		1		moral>nonmoral	
Heekeren	2003		8		9		moral>semantic judgment	
Heekeren	2005		12		8		moral>semantic judgment	
Moll	2001		10		10		moral>factual judgments	
Moll	2002a		7		3		moral>nonmoral judgment	
Parkinson	2011		38		6		disgust+harm+dishonest>nonmoral judgments	
Prehn	2008		23		13		socio-normative judgments>nonmoral judgments	
Reniers	2012		24		9		moral>nonmoral judgment	
Schleim	2010		40		6		moral>nonmoral jugdments	
Sinke	2010		14		9		threat>tease jugdments	
Sommer	2010		12		6		moral conflict>nonmoral conflict	
Takahashi	2008		15		2		moral depravity>nonmoral	
**First Author**	**Year**	**n**		**Foci**		**Contrast**		**Passive**
Akitsuki	2009	26		23		self+other pain>self		
Basile	2011	22		3		deontological+altruistic guilt>nonmoral emotions		
Berhotz	2002	12		18		intentional violation of social norms>social behaviors		
Borg	2008	15		24		(pathogen+incest+nonsexual) moral>neutral recall		
Decety	2011	22		10		imagine hurt>help		
Finger	2006	16		5		moral>social transgression+neutral		
Harenski	2006	30		7		moral>neutral		
Harenski	2010	14		9		moral>neutral		
ImmordinoYang	2009	13		9		admiration for virtue>neutral		
Kedia	2008	35		5		moral emotions>neutral		
Luo	2006	20		9		moral (illegal)>neutral		
Mercadillo	2011	24		9		compassion>objects+scenes		
Michl	2012	14		19		guilt>neutral		
Moll	2002b	7		12		moral>neutral		
Moll	2005	13		16		moral (indignation)>neutral		
Robertson	2007	16		5		moral (care,justice)>neutral (strategic,tactical)		
Takahashi	2004	19		5		guilt>neutral		
Wagner	2011	15		24		guilt>neutral		

After a systemic review of stimulus properties and experiment designs, we determined the two broad categories, labeled “Active” and “Passive”, that were distinguished based upon the presence or absence of a behavioral response related to the moral content. The Active category was marked by the presence of this overt response in which participants were aware of, and attended to, the moral content of the stimulus they were presented with. Participants were explicitly instructed to make a judgment related specifically to the moral content. Examples for the instruction are, “Is the action morally appropriate?”; “Would you do X?”; “Please rate the permissibility of the moral action.” For the Active studies, 22 experiments involving 439 participants and 186 foci were found. Our second category involved “Passive” moral task conditions. This category involved studies in which the participants were not explicitly asked to give a behavioral response that related to the moral content. In these tasks the behavioral rating was incidental to the experimental question of interest. For example, participants answered, “Does the image presented involve a living object?’. In total, 18 experiments involving 351 participants and 212 foci fulfilled the Passive criteria. In active tasks, activity during hypothetical moral judgments was contrasted with activity during non-moral judgments. In passive tasks, moral stimuli were typically contrasted with neutral or non-moral emotional stimuli. Parallel to the experimental design, in active tasks the analysis was time-locked to judgment response, whereas in passive tasks the analysis was either time-locked to stimulus presentation or motor response. Moreover, to rule out the possibility that studies in the active category were disproportionately based on text-based stimuli, we categorized the studies in Active and Passive categories into two groups based on stimulus modality used, i.e. visual vs. text-based and conducted a chi-square test. Results did not reveal statistically significant difference between the two conditions, *X* ^2^ (1, 40) = 2.16, p = 0.145 suggesting that stimulus modality was unlikely to disproportionately effect the results.

ALE is a coordinate based random effects meta-analysis method that determines reliable patterns of brain activity across several independent neuroimaging studies [26], [27]. Each statistically significant activation location is modeled as a 3D Gaussian probability distribution centered at the corresponding peak coordinate and a series of permutation tests are applied to differentiate true convergence from random clustering. The permutation procedure ensures that the distribution reflects a random spatial association between experiments and regards the distribution of foci within each experiment as fixed. This yields an ALE score that reflects the likelihood of a given voxel’s activation across all studies. Then, those ALE scores obtained under the null distribution are tested against the true ALE scores. Since larger sample sizes were modeled by using smaller kernels, the algorithm accounted for variance between subjects. The analysis was restricted to voxels with a grey matter probability exceeding 10%.

All study coordinates were entered in GingerALE 2.1 in the stereotaxic space of Montreal Neurological Institute (MNI). Studies that reported their coordinates using Talairach and Tourneaux space were transformed into MNI space using icbm2tal transform [26]. To determine reliable activity across studies using active moral judgment tasks, we conducted an ALE analyses with pooled foci from studies that fall under the Active category. Then, to determine reliable activity across studies using passive moral tasks, we conducted another ALE analysis with pooled foci from studies that fall under the Passive category. Conjunction and contrast analyses were also calculated using ALE subtraction analysis in order to determine common and distinct activation clusters associated with the moral cognition tasks. This analysis accounts for differences in sample size and is restricted to those voxels that show an effect in the Active and Passive meta-analyses. All reported contrasts were thresholded at a corrected *p*<.05 and cluster level threshold was set to 175 mm^3^.

## Results

**Figure 2:**
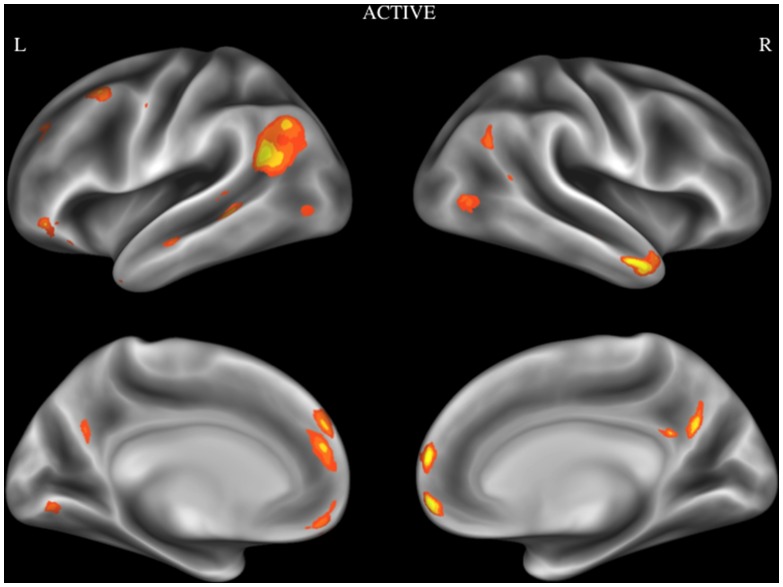
Activation likelihood estimation map showing significant convergent activity for studies in the active category. Surface maps of the activation likelihood clusters (false discovery rate *P*<.05) are shown on an inflated surface map in Caret [60].

In order to examine reliable activity during deliberative moral reasoning, an ALE analysis was performed using activation foci obtained from studies in the Active category. Brain regions that show consistent activation during active moral judgment tasks were the temporoparietal junction (TPJ), medial prefrontal cortex (MPFC), ventromedial prefrontal cortex (VMPFC), temporal pole/middle temporal gyrus, posterior cingulate cortex (PCC), dorsomedial prefrontal cortex (dMFC), middle frontal gyrus, angular gyrus, posterior parts of middle temporal gyrus, inferior frontal gyrus (IFG), lingual gyrus, superior temporal sulcus (see Figure 2). Coordinates of the activation maxima and cluster size are given in Table 2.

**Table 2:** Brain regions showing consistent activation during active moral judgment tasks.

	Anatomical Label	Cluster Volume	MNI Coordinates (X,Y,Z)		
L	TPJ	5008	−48	−56	22
L	MPFC	2488	−6	46	20
R	VMPFC	2016	4	58	−8
R	Temporal pole (middle temporal gyrus)	1832	54	8	−28
R/L	PCC	1368	0	−58	30
L	dMPFC	1208	−2	52	32
L	Middle frontal gyrus	984	−38	14	52
R	Angular gyrus	976	54	−66	34
L	Posterior middle temporal gyrus	816	−58	−42	0
L	IFG	568	−46	38	−12
L	Anterior superior frontal sulcus	312	−30	38	34
R	Inferior temporal sulcus	264	46	−66	2
L	Lingual gyrus	240	−6	−76	−2
L	IFG	184	−42	26	−20
L	Superior temporal sulcus	176	−56	−14	−14

Captions: TPJ = temporal parietal junction, MPFC = medial prefrontal cortex, VMPFC = ventromedial prefrontal cortex, PCC = posterior cingulate cortex, dMPFC = dorsomedial prefrontal gyrus, IFG = inferior frontal gyrus.

**Figure 3:**
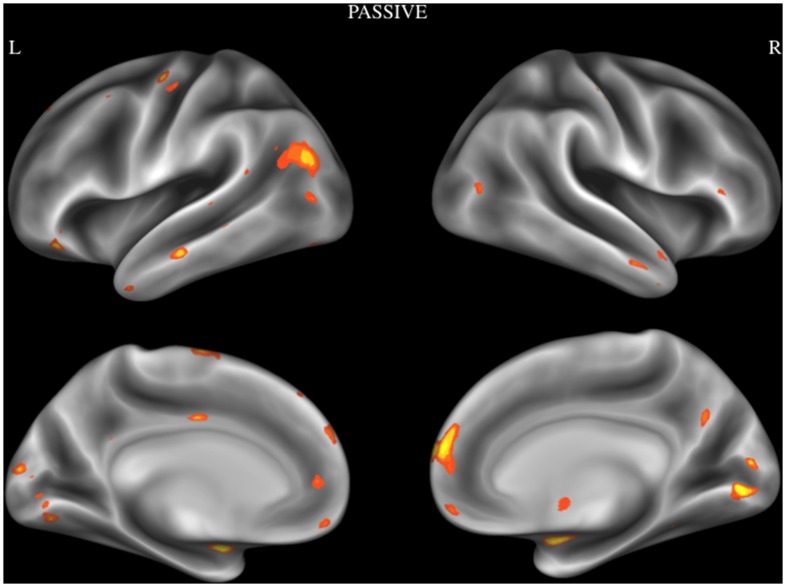
Activation likelihood estimation map showing significant convergent activity for studies in the passive category. Surface maps of the activation likelihood clusters (false discovery rate *P*<.05) are shown on an inflated surface map in Caret [60].

In order to examine reliable activity patterns associated with emotional moral processing, an ALE analysis was performed using activation foci from Passive category. Brain regions that showed reliable activation during passive tasks were dMPFC, lingual gyrus, amygdala, TPJ extending to posterior superior temporal sulcus, VMPFC, superior temporal sulcus, temporal pole, IFG, supplementary motor area, cuneus, precentral gyrus, PCC, medial temporal gyrus, middle cingulate cortex, and the mammillary body. See Figure 3 and Table 3.

**Table 3:** Brain regions showing consistent activation during passive tasks.

	Anatomical Label	Cluster Volume	MNI Coordinates (X,YZ)		
L	dMPFC	3184	−2	60	32
R	Lingual gyrus	1568	4	−82	4
L	Amygdala	824	−22	−6	−16
R	Amygdala	816	24	−4	−14
L	TPJ/posterior superior temporal sulcus	744	−44	−72	24
R	VMPFC	600	2	54	−10
L	Superior temporal sulcus	560	−62	−12	−12
R	Temporal pole	472	54	6	−22
L	IFG	336	−42	32	−14
L	Supplementary motor area	320	−12	−10	70
L	Lingual gyrus	304	−10	−80	−8
L	Cuneous	296	−4	−94	14
L	Precentral gyrus	296	−42	−8	60
L	Anterior temporal pole	288	−48	10	−42
R\L	Posterior cingulate gyrus	232	0	−62	32
L	Middle temporal gyrus	224	−68	−36	0
L	Middle cingulate cortex	200	−2	−10	40
R	Mammillary Body	184	4	−4	−6

Captions: dMPFC = dorsomedial prefrontal gyrus, TPJ = temporal parietal junction, VMPFC = ventromedial prefrontal cortex, IFG = inferior frontal gyrus.

**Figure 4:**
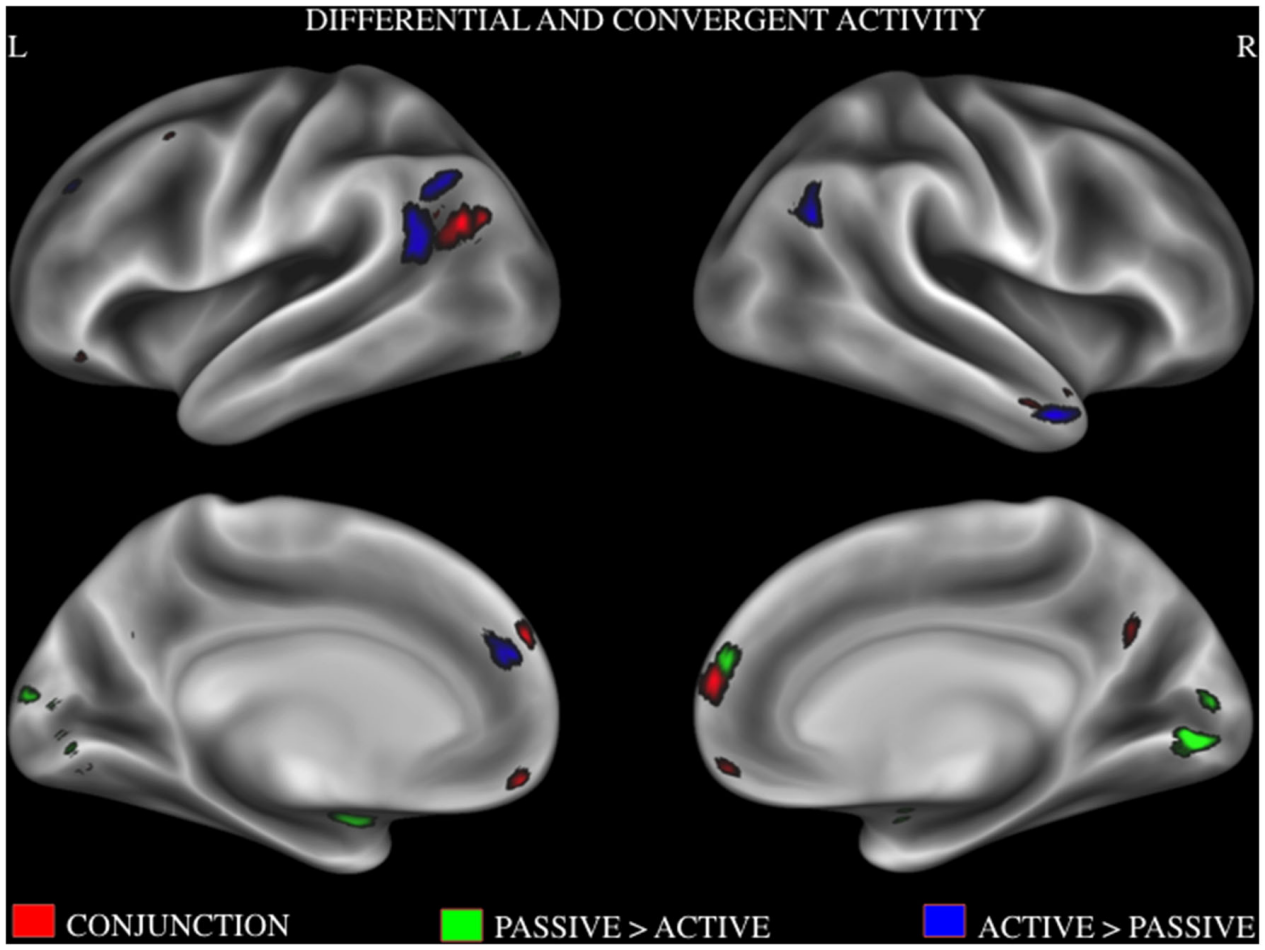
Combined activation likelihood estimation map showing significant activation clusters. red = active >passive; green = conjunction of active and passive; blue = passive>active. Surface maps of the activation likelihood clusters (false discovery rate *P*<.05) are shown on an inflated surface map in Caret [60].

To determine convergent activity between those active and passive tasks, a conjunction analysis was performed using activation maps that we obtained from the above ALE analyses. Brain regions that show consistent activity patterns during both active and passive tasks were dMPFC, PCC, temporal pole, VMPFC, TPJ and IFG. To determine divergent activity between the two moral tasks, a contrast analysis was performed. Brain regions that showed reliable activity during active compared to passive tasks were superior and anterior regions of TPJ, angular gyrus, temporal pole, and left MPFC. Brain regions that showed reliable activity during passive tasks compared to active tasks were the lingual gyrus, left amygdala and right MPFC. See Figure 4 for convergent and dissociated activity patterns across the two conditions and Table 4 for activation maxima and cluster sizes.

**Table 4:** Result of contrast analysis and conjunction analysis.

	C#		Anatomical Label	Cluster Volume	MNI Coordinates (X,Y,Z)		
**Result of contrast** **analysis Active >Passive**	1	L	TPJ	1720	−54	−68	34
	2	R	Angular gyrus	728	56	−62	26
	3	R	Temporal pole	392	52	12	−32
	4	L	Medial prefrontal cortex	304	−8	42	24
**Results of contrast** **analysis Passive>Active**	1	R\L	Lingual gyrus	1384	0	−86	−2
	2	L	Amygdala	552	−24	−6	−20
	3	R	Medial prefrontal cortex	320	2	58	24
**Results of conjunction** **analysis**	1	R	Medial prefrontal cortex	648	6	58	16
	2	R\L	PCC	168	0	−60	31
	3	R	Temporal Pole	176	54	7	−23
	4	R\L	VMPFC	520	−0.02	58	−11
	5	L	Medial prefrontal cortex	176	−3	55	34
	6	L	TPJ	56	−48	−71	24
	7	L	IFG	24	−44	34	−14

TPJ = temporal parietal junction, PCC = posterior cingulate cortex VMPFC = ventromedial prefrontal cortex, IFG = inferior frontal gyrus.

## Discussion

Investigations into the neural correlates of morality have centered upon active and explicit moral deliberations in addition to indirect, passive exposure to morally laden stimuli. In this study, we examined reliable patterns of brain activity associated with these two categories of moral cognition tasks. In addition to examining task-specific effects, we conducted a conjunction analysis to identify the neural correlates of moral cognition that are independent of these specific task demands, and thus putatively more associated with moral processing. This conjunction analysis revealed reliable activity within the default network of the brain. In a direct comparison between active and passive task conditions, dissociable patterns of brain activity were observed that likely reflect task context and specific temporal dynamics of task structure during moral information processing.

Both active and passive moral cognitive tasks engage core structures of the default network [18], [20], [28], [29], including MPFC and PCC. The default network is consistently involved in self-generated thought, such as generating predictions related to the contextual features of the stimuli [19], [30] in addition to engaging in spontaneous or deliberative mental state attribution [31]. MPFC is involved in appraisal of self-relevant information but has dissociable functions. Ventral aspects of MPFC, together with subcortical structures, have been associated with feature-based appraisal, specifically detection of and orientation to self-relevant content. Dorsal aspects of MPFC have been associated with introspective appraisal of self-relevance [32], [33], an important determinant of moral behavior [8]. The temporal pole, also reliably identified in the conjunction analysis, is involved in representing and retrieving social knowledge [34]. Reliable clusters of activation were also observed in the TPJ. This region has recently been implicated in the combination of convergent streams of information to construct a social context for behavior, which depends on information from internal goals, memory, and semantic abstraction [35]. IFG activity, which is often associated with social perception, recognition of emotional faces, and intentional motion, was also reliable [36], [37]. The pattern of overlapping activation clusters associated with both active and passive tasks is aligned with other qualitative reviews of fMRI studies on moral cognition [2], [7], [38]. These data are also consistent with findings of a previous meta-analysis that demonstrated convergent activity in brain areas engaged in theory-of-mind and empathy [39]. We suggest that core features include social and contextual information processing during both active moral judgments and passive viewing of moral stimuli.

Active moral cognitive tasks, such as moral dilemmas, are marked by the presence of an explicit decision. When participants make an explicit decision, they engage in a deliberative reasoning process that involves an allocation of attention to internal resources such as memory, simulation, mentalizing and conflict monitoring. This deliberation is further accompanied by increased associative process of integrating the information. Contrast analyses between active versus passive tasks revealed differential activity in superior and anterior parts of TPJ, angular gyrus and right temporal pole. Activity in dorsomedial frontal regions was lateralized to left hemisphere during active tasks, compared to the right lateralization during passive tasks. Functional specialization of conceptual and perceptual cognition to the left and right hemispheres, respectively, may be an organizing principle of the brain [40]. Based on this, we propose that this lateralization is attributable to the increased demand for conceptual processing during active tasks. TPJ activity within moral cognition is usually interpreted as reflecting spontaneous inference about the mental state of others [41]. The TPJ also functions as a hub, integrating information from several domains such as attention, memory and semantic abstraction creating a social context especially when there is a decision situation that involves another agent [42]. Differential activity in superior and anterior parts of TPJ in active tasks is in line with this interpretation. The temporal pole is associated with the processing of personally relevant social information. The right anterior temporal pole in particular is responsible from linking high-level sensory representations with emotional responses and social memory [43]. Another region, angular gyrus has been argued to function as a cross-modal hub where multisensory information is integrated for comprehension, manipulation of mental representations, and reorienting attention to relevant information [44]. We hypothesize that these regions, acting in concert with the core regions discussed above, are involved in the deliberative process of moral reasoning and reflect the retrieval of social knowledge from memory, mental state attribution, and the construction of social context through an associative process.

When incidentally or passively perceiving moral stimuli, the moral salience of the information is spontaneously processed [16]. This is observable at the level of sensory information processing. Passive moral tasks, compared with active tasks, show reliable activity in areas associated with visual and emotional information processing including the lingual gyrus, and the left amygdala. The amygdala responds to many forms of visual emotional stimuli and is involved in the detection of innate, biologically and socially relevant information [45](Sergerie, 2008). It has projections to visual cortex and modifies attention to visual information in the ventral stream, including lingual gyrus [46], [47]. Right-lateralized activity in dMPFC during passive tasks compared to active tasks supports our interpretation that the passive tasks are associated with increased demand for perceptual processing (as opposed to left lateralized conceptual processing; e.g. [40]). Divergent activity between active and passive moral tasks is consistent with the existence of multiple moral processing types that are either related with quick affective responses or deliberative reasoning (e.g. [6]–[8]).

One critical driver of the neural differences associated with active and passive moral cognitive tasks are the temporal dynamics of the experimental designs used to assess moral cognition. Active and passive tasks differ in the time-locked hemodynamic response window of analysis. Active moral cognitive tasks usually target activity associated with the response judgment. In contrast, passive tasks target the stimulus presentation. Borg and colleagues compared deliberation-locked (time-locked to stimulus onset modeled with no specified duration as variable length boxcar function) and verdict-locked activity (time-locked to participant response) and found that the two models were associated with differential activity patterns [48]. Their results demonstrated that hemodynamic activity in VMPFC, PCC, and TPJ, were associated primarily with moral deliberation as opposed to moral verdicts. Moral verdicts, on the other hand, were associated with activity in anterior insula extending to inferior frontal gyri, temporal poles, basal ganglia and amygdala. Importantly, amygdala and basal ganglia activity disappeared when the averaged stimulus-related activity preceding and leading up to a decision was included in the model. These findings may inform the temporal unfolding of moral information processing and underline the importance of distinguishing between different moral task types. It is likely that passive and active tasks convey different aspects of the evolving temporal dynamics of brain activity during moral cognitive processing. In this view, early activity in the ventral visual stream and amygdala could flag salient moral information, working in concert with common regions in PCC, MPFC, TPJ and temporal poles. Depending on contextual demands, moral reasoning may be required and lead to the activation of bilateral TPJ, angular gyrus, additional regions in temporal poles and anterior cingulate cortex.

Default network recruitment during active tasks has been called into question by a recent study assessing more ecologically valid experimentation into moral cognition [49].While hypothetical moral decisions elicited activity in PCC and inferior parietal lobule, real moral decisions elicited activity in areas such as amygdala and anterior cingulate cortex. Moreover, researchers demonstrated an inconsistency between participants’ decisions during real and hypothetical moral situations in a separate behavioral study [50]. By systematically enhancing the contextual information available to participants, such as the salience of personal gain and thereby reducing the opportunity for mental simulation, they brought participants’ responses in line with their behavior in real situations. Default network activity observed in the present meta-analysis may therefore result from responding to limited contextual information and indicate neural activity related to mental simulations [22] and contextual associations [30]. Central structures of the salience network include dorsal anterior cingulate cortex and bilateral insula, as well the amygdala and other structures [51]. This network responds to behaviorally salient events and may be responsible for detection of morally salient events in the environment and signal subsequent modulation of activity in default and/or frontoparietal control networks [52]. Recent research provided evidence about the causal influences of salience network on default network activity during moral judgments [11] and emotion’s role in modulating other brain regions and alerting the individual to the moral salience of a situation [53]. We postulate that limited reliable activity in salience network regions in the present meta-analysis might be explained by temporal dynamics of neural response, the hypothetical nature of the stimuli and specifics temporal dynamics of the experimental tasks. The amygdala activity in passive tasks, captured early on, signals salience and consecutive recruitment of the salience network may modulate default network activity, depending on certain features of the task and moral stimuli. Future work will be necessary to assess default and salience network interactions in the apprehension of salient moral information, and the contextual associative processes involved in moral cognition and reasoning.

Theoretical attempts that describe morality vary in terms of the interaction between emotion and cognition or in terms of their emphasis on deliberate/inferential versus automatic/intuitive processes [7]. For instance Greene proposed a dual-process theory of moral judgment [54]. Accordingly, characteristically deontological moral judgments, that are usually associated with duties and rights, are driven by automatic emotional responses. On the other hand characteristically utilitarian or consequentialist moral judgments, that are associated with the conception of greater good, are driven by controlled cognitive processes. Evidence for this theory comes from neuroimaging studies in which utilitarian judgments are found to be associated with longer response times and with increased activation in areas implicated in deliberative processing; whereas deontological judgments were found to be associated with greater activation in areas related to affective processing. Kahane criticized this theory emphasizing that different modes of processing may not be attributable to different contents [55]. He argued that utilitarian judgments are counterintuitive in nature and the role of controlled processing is to sustain deliberation in those dilemma situations. On the other hand, an alternative view proposes that moral cognition requires integration of these mechanisms; such as the engagement of emotional states in conjunction with prospective thinking and representation of multiple outcomes of events and actions. According to this view, moral reasoning and emotion depend on associatively linked representations within fronto-temporo-limbic networks [7], [56]. The findings of this meta-analysis are in line with this interpretation and emphasize the role of associative processes during moral processing.

Recently the search for domain-specific neural modules gave its way to the discovery of large-scale, domain general networks that are distributed in structure and function [57]. Accordingly, psychological phenomena emerge through the spatiotemporal interaction of these networks. From this perspective, ‘emotional’ or ‘social’ phenomena are not attributable to specific neural regions, but arise from the interaction between regions and networks [58], [59].The findings of the present meta-analysis suggest that an investigation of moral behavior within the framework of large-scale brain networks, and their interactions, may yield fresh insight into the current theoretical debates.

## Supporting Information

Appendix S1 Studies included in the meta-analysis.
(DOCX)

Checklist S1 PRISMA 2009 Checklist.
(DOC)

Flow Diagram S1 PRISMA 2009 Flow Diagram.
(DOC)
