# Supplementary material for: Contextual and Perceptual Brain Processes Underlying Moral Cognition: A Quantitative Meta-Analysis of Moral Reasoning and Moral Emotions
Source: PLoS One. 2014 Feb 4;9(2):e87427. doi: 10.1371/journal.pone.0087427 (PMC3913597; doi:10.1371/journal.pone.0087427)
Supplement: Appendix S1 — Studies included in the meta-analysis. (DOCX) [file pone.0087427.s001.docx]

Studies included in the meta-analysis

Active/deliberative tasks

Avram, M., Gutyrchik, E., Bao, Y., Pöppel, E., Reiser, M., & Blautzik, J. (2012). Neurofunctional correlates of aesthetic and moral judgments. *Neuroscience letters*.

Bahnemann, M., Dziobek, I., Prehn, K., Wolf, I., & Heekeren, H. R. (2010). Sociotopy in the temporoparietal cortex: common versus distinct processes. *Social cognitive and affective neuroscience*, *5*(1), 48-58.

Borg, J. S., Hynes, C., Van Horn, J., Grafton, S., & Sinnott-Armstrong, W. (2006). Consequences, action, and intention as factors in moral judgments: An fMRI investigation. Journal of cognitive neuroscience, 18(5), 803-817.

Borg, J. S., Sinnott-Armstrong, W., Calhoun, V. D., & Kiehl, K. A. (2011). Neural basis of moral verdict and moral deliberation. *Social neuroscience*, *6*(4), 398-413.

Chiong, W., Wilson, S. M., D’Esposito, M., Kayser, A. S., Grossman, S. N., Poorzand, P., ... & Rankin, K. P. (2013). The salience network causally influences default mode network activity during moral reasoning. *Brain*, *136*(6), 1929-1941.

FeldmanHall, O., Dalgleish, T., Thompson, R., Evans, D., Schweizer, S., & Mobbs, D. (2012). Differential neural circuitry and self-interest in real vs hypothetical moral decisions. *Social cognitive and affective neuroscience*, *7*(7), 743-751.

FeldmanHall, O., Mobbs, D., & Dalgleish, T. (2013). Deconstructing the brain’s moral network: dissociable functionality between the temporoparietal junction and ventro-medial prefrontal cortex. *Social cognitive and affective neuroscience*.

Harada, T., Itakura, S., Xu, F., Lee, K., Nakashita, S., Saito, D. N., & Sadato, N. (2009). Neural correlates of the judgment of lying: A functional magnetic resonance imaging study. *Neuroscience research*, *63*(1), 24-34.

Harenski, C. L., Antonenko, O., Shane, M. S., & Kiehl, K. A. (2008). Gender differences in neural mechanisms underlying moral sensitivity. *Social cognitive and affective neuroscience*, *3*(4), 313-321.

Harrison, B. J., Pujol, J., López-Solà, M., Hernández-Ribas, R., Deus, J., Ortiz, H., ... & Cardoner, N. (2008). Consistency and functional specialization in the default mode brain network. *Proceedings of the National Academy of Sciences*, *105*(28), 9781-9786.

Hayashi, A., Abe, N., Ueno, A., Shigemune, Y., Mori, E., Tashiro, M., & Fujii, T. (2010). Neural correlates of forgiveness for moral transgressions involving deception. *Brain research*, *1332*, 90-99.

Heekeren, H. R., Wartenburger, I., Schmidt, H., Schwintowski, H. P., & Villringer, A. (2003). An fMRI study of simple ethical decision-making. *Neuroreport*, *14*(9), 1215-1219.

Heekeren, H. R., Wartenburger, I., Schmidt, H., Prehn, K., Schwintowski, H. P., & Villringer, A. (2005). Influence of bodily harm on neural correlates of semantic and moral decision-making. *Neuroimage*, *24*(3), 887-897.

Moll, J., Eslinger, P. J., & Oliveira-Souza, R. D. (2001). Frontopolar and anterior temporal cortex activation in a moral judgment task: preliminary functional MRI results in normal subjects. *Arquivos de neuro-psiquiatria*, *59*(3B), 657-664.

Moll, J., de Oliveira-Souza, R., Bramati, I. E., & Grafman, J. (2002). Functional networks in emotional moral and nonmoral social judgments. *Neuroimage*, *16*(3), 696-703.

Parkinson, C., Sinnott-Armstrong, W., Koralus, P. E., Mendelovici, A., McGeer, V., & Wheatley, T. (2011). Is morality unified? Evidence that distinct neural systems underlie moral judgments of harm, dishonesty, and disgust. *Journal of Cognitive Neuroscience*, *23*(10), 3162-3180.

Prehn, K., Wartenburger, I., Mériau, K., Scheibe, C., Goodenough, O. R., Villringer, A., ... & Heekeren, H. R. (2008). Individual differences in moral judgment competence influence neural correlates of socio-normative judgments. *Social Cognitive and Affective Neuroscience*, *3*(1), 33-46.

Reniers, R. L., Corcoran, R., Völlm, B. A., Mashru, A., Howard, R., & Liddle, P. F. (2012). Moral decision-making, ToM, empathy and the default mode network. *Biological psychology*, *90*(3), 202-210.

Schleim, S., Spranger, T. M., Erk, S., & Walter, H. (2011). From moral to legal judgment: the influence of normative context in lawyers and other academics. *Social cognitive and affective neuroscience*, *6*(1), 48-57.

Sinke, C. B. A., Sorger, B., Goebel, R., & de Gelder, B. (2010). Tease or threat? Judging social interactions from bodily expressions. *Neuroimage*, *49*(2), 1717-1727.

Sommer, M., Rothmayr, C., Döhnel, K., Meinhardt, J., Schwerdtner, J., Sodian, B., & Hajak, G. (2010). How should I decide? The neural correlates of everyday moral reasoning. *Neuropsychologia*, *48*(7), 2018-2026.

Takahashi, H., Kato, M., Matsuura, M., Koeda, M., Yahata, N., Suhara, T., & Okubo, Y. (2008). Neural correlates of human virtue judgment. *Cerebral Cortex*, *18*(8), 1886-1891.

Passive/emotional tasks

Akitsuki, Y., & Decety, J. (2009). Social context and perceived agency affects empathy for pain: an event-related fMRI investigation. *Neuroimage*, *47*(2), 722-734.

Basile, B., Mancini, F., Macaluso, E., Caltagirone, C., Frackowiak, R. S., & Bozzali, M. (2011). Deontological and altruistic guilt: Evidence for distinct neurobiological substrates. *Human brain mapping*, *32*(2), 229-239.

Berthoz, S., Armony, J. L., Blair, R. J. R., & Dolan, R. J. (2002). An fMRI study of intentional and unintentional (embarrassing) violations of social norms. *Brain*, *125*(8), 1696-1708.

Borg, J. S., Lieberman, D., & Kiehl, K. A. (2008). Infection, incest, and iniquity: Investigating the neural correlates of disgust and morality. *Journal of Cognitive Neuroscience*, *20*(9), 1529-1546.

Decety, J., & Porges, E. C. (2011). Imagining being the agent of actions that carry different moral consequences: an fMRI study. *Neuropsychologia*, *49*(11), 2994-3001.

Finger, E. C., Marsh, A. A., Kamel, N., Mitchell, D. G., & Blair, J. R. (2006). Caught in the act: the impact of audience on the neural response to morally and socially inappropriate behavior. *Neuroimage*, *33*(1), 414-421.

Harenski, C. L., & Hamann, S. (2006). Neural correlates of regulating negative emotions related to moral violations. *Neuroimage*, *30*(1), 313-324.

Harenski, C. L., Antonenko, O., Shane, M. S., & Kiehl, K. A. (2010). A functional imaging investigation of moral deliberation and moral intuition. *Neuroimage*, *49*(3), 2707-2716.

Immordino-Yang, M. H., McColl, A., Damasio, H., & Damasio, A. (2009). Neural correlates of admiration and compassion. *Proceedings of the National Academy of Sciences*, *106*(19), 8021-8026.

Kédia, G., Berthoz, S., Wessa, M., Hilton, D., & Martinot, J. L. (2008). An agent harms a victim: a functional magnetic resonance imaging study on specific moral emotions. *Journal of Cognitive Neuroscience*, *20*(10), 1788-1798.

Luo, Q., Nakic, M., Wheatley, T., Richell, R., Martin, A., & Blair, R. J. R. (2006). The neural basis of implicit moral attitude—an IAT study using event-related fMRI. *Neuroimage*, *30*(4), 1449-1457.

Mercadillo, R. E., Díaz, J. L., Pasaye, E. H., & Barrios, F. A. (2011). Perception of suffering and compassion experience: brain gender disparities. *Brain and cognition*, *76*(1), 5-14.

Michl, P., Meindl, T., Meister, F., Born, C., Engel, R. R., Reiser, M., & Hennig-Fast, K. (2012). Neurobiological underpinnings of shame and guilt: A pilot fMRI study. *Social cognitive and affective neuroscience*.

Moll, J., de Oliveira-Souza, R., Eslinger, P. J., Bramati, I. E., Mourão-Miranda, J., Andreiuolo, P. A., & Pessoa, L. (2002). The neural correlates of moral sensitivity: a functional magnetic resonance imaging investigation of basic and moral emotions. *The Journal of Neuroscience*, *22*(7), 2730-2736.

Moll, J., de Oliveira-Souza, R., Moll, F. T., Ignácio, F. A., Bramati, I. E., Caparelli-Dáquer, E. M., & Eslinger, P. J. (2005). The moral affiliations of disgust: A functional MRI study. *Cognitive and behavioral neurology*, *18*(1), 68-78.

Robertson, D., Snarey, J., Ousley, O., Harenski, K., Bowman, F. D., Gilkey, R., & Kilts, C. (2007). The neural processing of moral sensitivity to issues of justice and care. *Neuropsychologia*, *45*(4), 755-766.

Takahashi, H., Yahata, N., Koeda, M., Matsuda, T., Asai, K., & Okubo, Y. (2004). Brain activation associated with evaluative processes of guilt and embarrassment: an fMRI study. *Neuroimage*, *23*(3), 967-974.

Wagner, U., N'Diaye, K., Ethofer, T., & Vuilleumier, P. (2011). Guilt-specific processing in the prefrontal cortex. *Cerebral cortex*, *21*(11), 2461-2470.
