# Supplementary material for: Contextual and Perceptual Brain Processes Underlying Moral Cognition: A Quantitative Meta-Analysis of Moral Reasoning and Moral Emotions
Source: PLoS One. 2014 Feb 4;9(2):e87427. doi: 10.1371/journal.pone.0087427 (PMC3913597; doi:10.1371/journal.pone.0087427)
Supplement: Flow Diagram S1 — PRISMA 2009 Flow Diagram. (DOC) [file pone.0087427.s003.doc]

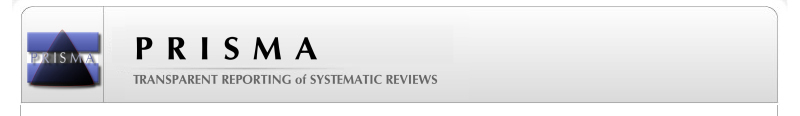
**PRISMA 2009 Flow Diagram**

**Screening**

**Included**

**Eligibility**

**Identification**

Records identified through database searching
(n = 2694 )

Additional records identified through other sources
(n = 19 )

Records after duplicates removed
(n = 451 )

Records screened
(n = 451 )

Records excluded
(n = 365 )

Full-text articles assessed for eligibility
(n = 86 )

Full-text articles excluded, with reasons
(n = 44 )

Studies included in qualitative synthesis
(n = 42 )

Studies included in quantitative synthesis (meta-analysis)
(n = 40 )
